# Habitat Use and Selection by Giant Pandas

**DOI:** 10.1371/journal.pone.0162266

**Published:** 2016-09-14

**Authors:** Vanessa Hull, Jindong Zhang, Jinyan Huang, Shiqiang Zhou, Andrés Viña, Ashton Shortridge, Rengui Li, Dian Liu, Weihua Xu, Zhiyun Ouyang, Hemin Zhang, Jianguo Liu

**Affiliations:** 1 Center for Systems Integration and Sustainability, Department of Fisheries and Wildlife, Michigan State University, East Lansing, MI, United States of America; 2 Key Laboratory of Southwest China Wildlife Resources Conservation, China West Normal University, Ministry of Education, Nanchong, China; 3 China Conservation and Research Center for the Giant Panda (CCRCGP), Wolong Nature Reserve, Sichuan, China; 4 Department of Geography, Michigan State University, East Lansing, MI, United States of America; 5 State Key Laboratory of Urban and Regional Ecology, Research Center for Eco–environmental Sciences, Chinese Academy of Sciences, Beijing, China; Sichuan University, CHINA

## Abstract

Animals make choices about where to spend their time in complex and dynamic landscapes, choices that reveal information about their biology that in turn can be used to guide their conservation. Using GPS collars, we conducted a novel individual-based analysis of habitat use and selection by the elusive and endangered giant pandas (*Ailuropoda melanoleuca*). We constructed spatial autoregressive resource utilization functions (RUF) to model the relationship between the pandas' utilization distributions and various habitat characteristics over a continuous space across seasons. Results reveal several new insights, including use of a broader range of habitat characteristics than previously understood for the species, particularly steep slopes and non-forest areas. We also used compositional analysis to analyze habitat selection (use with respect to availability of habitat types) at two selection levels. Pandas selected against low terrain position and against the highest clumped forest at the at-home range level, but no significant factors were identified at the within-home range level. Our results have implications for modeling and managing the habitat of this endangered species by illustrating how individual pandas relate to habitat and make choices that differ from assumptions made in broad scale models. Our study also highlights the value of using a spatial autoregressive RUF approach on animal species for which a complete picture of individual-level habitat use and selection across space is otherwise lacking.

## Introduction

The relationship between animals and their habitats is a central component of wildlife ecology [1]. One important area of research involves understanding the behavior of individual animals as they use habitats distributed across heterogeneous space [2, 3]. Such studies often reveal a wealth of information that cannot be obtained by population-level surveys, including information on fine-scale variation over space and time [2, 4]. Research has also been extended to the study of habitat selection, or the choice of habitats relative to their availability on the landscape [5, 6]. Such studies can inform conservation efforts of endangered species by revealing the full range of resources used that may be missed by population surveys alone, while also pinpointing which types of habitats are selected in higher proportion to their availability, thus potentially warranting increased conservation focus [7, 8].

This constitutes an important research topic for endangered species such as the giant panda (*Ailuropoda melanoleuca*). Endemic to mountainous forests found in southwestern China, giant pandas are the most endangered ursid on earth. Currently limited to a mere 25,000 km^2^ of estimated suitable habitat [9], the estimated 1,864 remaining giant pandas are facing human threats including road construction, timber harvesting, tourism, and livestock grazing [9, 10]. The remaining panda habitat is defined by the existence of bamboo, their main food source, which makes up over 99% of their diet [11]. Bamboo occurs in mixed deciduous and coniferous forests in areas that are often rugged, with steep mountainsides and rapidly changing elevation [12, 13].

Aside from bamboo, panda habitat suitability is most commonly defined by three variables- forest, slope, and elevation [14]. Pandas use forests located at the mid-elevations that provide suitable conditions for bamboo growth [11, 15]. Slope is also one of the most important habitat characteristics for pandas, as pandas use areas of low or moderate slope for energetically efficient traveling [11, 16]. Pandas also use areas with high solar radiation, choosing warmer topographic aspects [17] in addition to areas farther from focal areas of human activity such as villages [18] and in areas not recently subjected to timber harvesting [13, 19]. Pandas also select many of these same characteristics at a higher proportion than what is available (e.g. higher bamboo cover and higher solar radiation) or at a lower proportion than what is available (e.g. steep slopes) [20].

Despite this information, many gaps remain in our understanding of the habitat use and selection by this elusive species [20, 21]. Previous research was mainly conducted at the population level, with little known about habitat use and selection of individual giant pandas. This is in large part due to a government moratorium on all giant panda telemetry from 1995–2006 [22, 23], which limited the information available on the behavior of individual pandas. As a result, information on habitat use and selection was derived from panda signs (e.g., fecal droppings) detected along transects sampled throughout their habitat areas. Such an approach, while valuable, leaves little appreciation for variation in intensity of habitat use and selection because it is derived as a binary (presence/non-presence) variable and cannot be ascribed to individual animals. This limitation started to be overcome in a recent study by Zhang et al. [24] involving habitat use of GPS-collared giant pandas which found significant effects of several geophysical factors (including elevation and slope).

But several questions remain to be answered, including the relationship between habitat use and both existing habitat suitability models and habitat selection. For instance, existing habitat suitability models were built from wildlife sign data, which does not capture the complete picture of areas that pandas use less frequently but may still be valuable. Such population level data, while valuable, may also be biased if collected in a non-random manner, such as on easily accessible trails, as is common in panda research [20]. The proportion of individual panda home ranges made up of different habitat suitability classes is unknown, as is whether some variables may be less important when combined with other variables in a multivariate model of habitat use. In addition, there has only been one individual-level assessment of habitat selection in the species [17, 25]. The individual-level analysis is important because it sheds light on how each panda is making choices on the landscape in relationship to what type of resources are available to it. This was a valuable work, but was derived from pairing radio telemetry locations (with limited spatial accuracy and temporal resolution) to other locations in a reserve outside of the home ranges, leaving remaining questions about variation in habitat use across the home range and definition of available habitats.

To begin filling these knowledge gaps, in this study we examined the habitat use and selection by individual, GPS-collared giant pandas across space and time. Our objectives were to (1) analyze the biogeophysical factors related to continuous predictions of habitat use via a multivariate spatial autoregressive modeling approach, (2) relate habitat use to existing habitat suitability models for giant pandas via spatial overlay techniques, and (3) investigate habitat selection (use/availability) by individual giant pandas at within-home range and at-home range selection levels via a modified compositional analysis. This study generates new information on the ecology of this endangered species, specifically by providing necessary individual context for understanding how pandas relate to their complex environments, in turn informing conservation by prioritizing specific areas of remaining panda habitat that need to be managed.

## Methods

### Study area and panda subjects

The study area is located in Wolong Nature Reserve (102°52’– 103°24’E, 30°45’– 31°25’N), Sichuan, China. Home to around 10% of the total wild giant panda population [26], the reserve contains ample forest stretching across mountains with steep slopes (above 50°, [11]). Our team has been doing research in the reserve since 1996 [27–34]. Many of the results and methods generated in the reserve have also been applied to regional, national, and global levels [35–44]. It is our hope that this study will also be useful for other species in other parts of the world.

**Fig 1:**
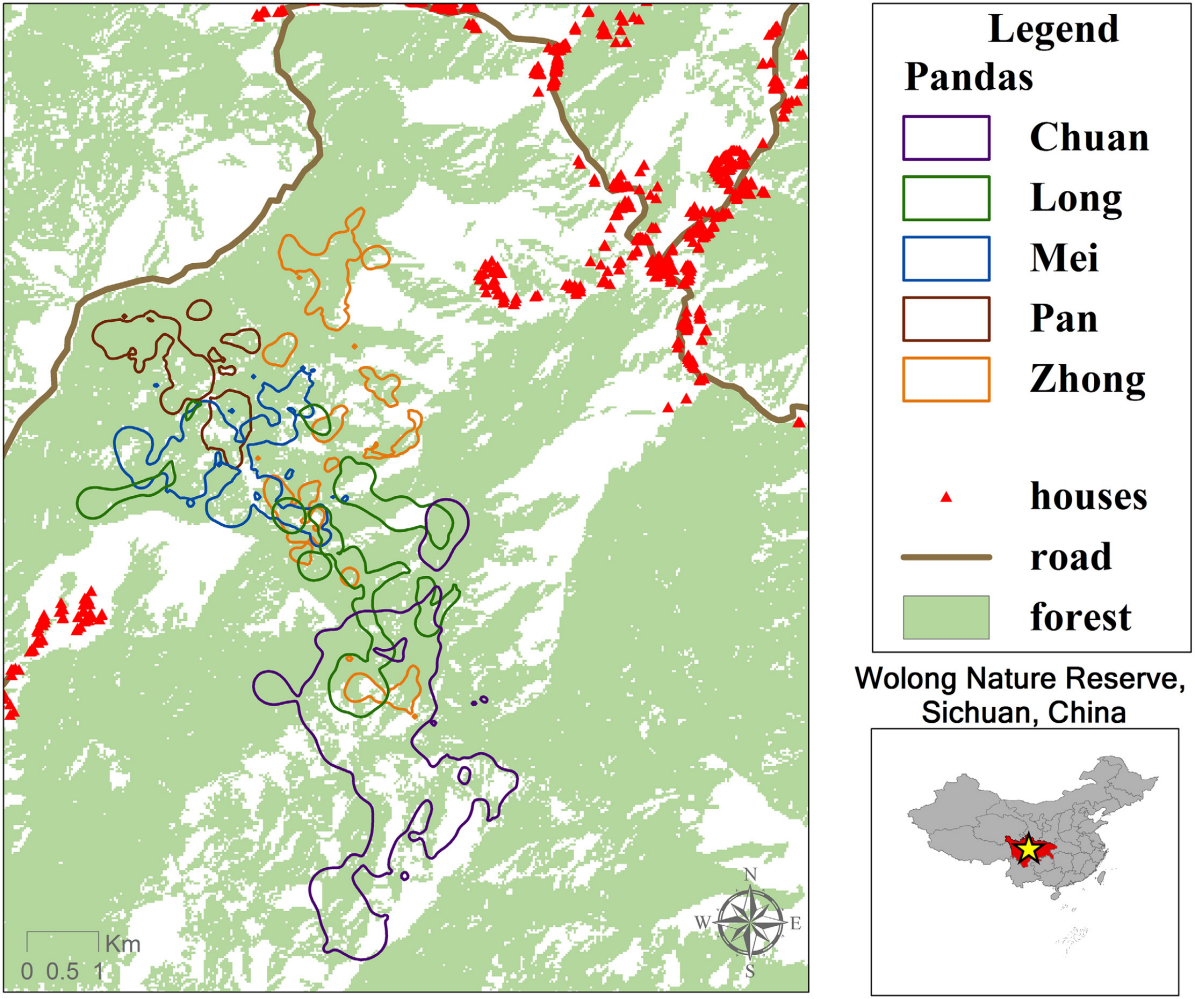
Hetaoping study area for giant panda GPS collar research in Wolong Nature Reserve, China. Forest layer is derived from supervised classification of Landsat TM imagery [43]. Chinese names refer to individual pandas.

The study was conducted in the northeastern portion of the reserve in an area known as Hetaoping (Fig 1). Roughly 40 km^2^ in size and spanning an elevational range of 1,800 to 3,100 m, the study area includes mixed deciduous and coniferous forests and subalpine coniferous forests with bamboo dominating their understories. Common tree species include Chinese walnut (*Juglans cathayensis*), mono maple (*Acer mono*), hemlock (*Tsuga longibracteata*) and spruce (*Picea asperata*), while the main bamboo species are arrow (*Bashania fangiana*), umbrella (*Fargesia robusta*) and Yushan (*Yushania bravipaniculata*) bamboo.

Camera trapping and genetic testing of DNA extracted from field-collected feces suggest that Hetaoping supports a local population of 16–25 pandas [45]. Camera trapping also revealed the presence of other large mammals, including the tufted deer (*Elaphodus cephalophus*), serow (*Capricornis milneedwardsii)*, and sambar (*Rusa unicolor)*, although none are believed to be direct competitors with pandas for space and food [11]. Hetaoping is geographically bounded by human activity zones: it is located between two villages to the southwest and northeast and a provincial highway to the west (Fig 1).

Five giant panda individuals were captured in 2010 and 2011 at Hetaoping, fitted with GPS collars, and released (Table 1). Capture was accomplished using anesthetization dart guns loaded with weight-dependent doses of ketamine. Animals were handled for short (~30 minute) periods. Staff members at the China Conservation and Research Center for the Giant Panda (CCRCGP) were responsible for animal safety. Research was approved (via granting a waiver) by the Michigan State University Institutional Animal Care and Use Committee (IACUC). The study pandas included 4 females and 1 male, all adults except for a sub-adult female.

**Table 1:** Summary of study pandas and GPS collar performance over the one year period included in this study.

	Pan	Long	Mei	Zhong	Chuan
Sex	female	female	female	female	male
Age	adult	sub-adult	adult	adult	adult
Start date	4/18/2010	4/11/2011	4/18/2010	4/11/2011	4/11/2011
Days monitored	219	184	365	365	351
Total fixes recorded	507	458	961	458	1473
Fix acquisition rate	0.39	0.41	0.47	0.30	0.70

Pandas were fitted with Lotek GPS_4400 M collars (Lotek Engineering Inc., Newmarket, Ont., Canada). The collars weighed about 1.2 kg and recorded longitude, latitude, and elevation once every four hours. Collars also measured activity (movement of pandas' heads along the X and Y axes every 5 minutes). Number of days of monitoring varied across individuals (Table 1) due to either the collar falling off of the animal or collar damage. Data collected within one week of an individual's release were excluded to minimize bias introduced by the capture/release event. Static testing on collars in various habitat locations prior to deployment on pandas revealed a fix acquisition rate of over 90% for each collar (n = 30 habitat locations). Fix acquisition was not correlated to habitat characteristics (e.g., slope or forest cover). Fix acquisition rate of collars while worn by pandas was lower (30–70%, Table 1). This is likely due to animal behavior (e.g. antenna obstruction while the panda was sleeping or eating). A successful fix occurred at least once every 10 days (and usually at least once every 3 days) for all pandas except the male, whose collar malfunctioned and did not record data for two longer periods (11/14/2011-12/25/2011 and 3/27/2012-4/11/2012). While the overall higher fix acquisition rate of the male panda compared to the others may influence model results and performance, we did not see consistent patterns that differentiated this individual panda’s habitat use and selection data from that of the others. Positional errors of collars compared to a differentially corrected GPS unit averaged 16 to 23 m across individuals (n = 30 locations per collar). Since pre-deployment testing showed no significant difference in location error of 2-D (3 satellites) versus 3-D (4 or more satellites) locations, we included both in the analysis. However, we excluded data for which the elevation estimate was inaccurate (measuring below 1,000 m, n = 11% of all observations), as these fixes appeared to also have inaccurate longitude and latitude measurements.

### Estimating panda utilization distributions

A utilization distribution (UD) is a probability density function representing a continuous prediction of an animal's frequency of use across space [46]. We estimated the UD of each individual by applying the biased random bridge (BRB) movement model to the GPS locations [47]. We chose this model over another commonly used method, the Brownian bridge movement model (BBMM), because a comparison of the two approaches using the area under the receivers operating characteristic curve (AUC) approach (as in [48]) showed the BRB to outperform the BBMM model. The BRB model is a is a stochastic model composed of a biased random walk in which the probability of being found at a particular location is dependent on the starting and ending locations and the time elapsed between them. An advection-diffusion component accounts for animals having a higher probability of drifting toward certain directions. We estimated the probability density function using a circular bivariate normal distribution. The diagonal of the variance-covariance matrix of this model was the diffusion coefficient (*D*) [47]. Parameters *Tmax* (maximum step duration), *hmin* (location uncertainty parameter), and *Lmin* (minimum distance between successive locations) were set as 36 hours, 10 m, and 20 m, respectively. We used a standard diffusion parameter (*D*) chosen using the plug–in method by taking the average across all animals (*D* = 0.85 m^2^/s, [47]). We also used the collars’ activity data to correct for “active” time since the previous location (i.e. only the proportion of time with nonzero activity measured using the collars' activity sensors, [47]). We defined the extent of space use by each animal at the 95% UD boundary (commonly defined as the animal's home range [49]). For the purposes of this study and to standardize across individuals, we limited the data range to one year for those pandas with more than one year of data (Table 1; the second year of data was used for the adult female panda named Mei Mei due to a pregnancy in the first year). Home ranges of the individuals studied ranged from 1.2 to 6 km^2^ during this time period [23].

### Habitat characteristics

We examined 6 habitat characteristics relevant for habitat use of giant pandas. These included slope, elevation, terrain position, solar radiation, forest presence, and forest clumpiness. Slope, elevation, and forest presence are commonly used in giant panda habitat suitability mapping for the species. Pandas are believed to use gentle slopes due to ease of travel and mid-elevation forested areas due to suitability for bamboo growing conditions [14, 15]. Topographic position has been hypothesized to be an important predictor of panda use in the past, with pandas using ridges intensively for travel and scent communication [50, 51]. Solar radiation has been hypothesized to be an important predictor of panda use, with pandas using warmer areas more intensively than cooler areas [17].

Slope, elevation, topographic position, and solar radiation were derived from a Digital Elevation Model (DEM) released in 2011 by the National Aeronautics and Space Administration’s (NASA) Advanced Spaceborne Thermal Emission and Reflection Radiometer (ASTER GDEM v2, 29 m resolution). Topographic position was calculated using the topographic position index (TPI), a measure of the difference between the elevation in a pixel and the average elevation in the surrounding pixels (we chose a 9-pixel neighborhood area) calculated using the Land Facet Corridor Designer in ArcGIS [52]. Higher values represent mountain ridges and lower values represent valleys. Solar radiation was estimated using the Area Solar Radiation tool in ArcGIS (with a 200 m sky size and a year-long estimation using monthly intervals). The forest/non-forest layer was derived from a supervised classification (with an 82.6% accuracy) of Landsat TM imagery (30 x 30 m resolution) acquired in 2007 [53]. The measure of forest clumpiness we used was calculated on the Landsat forest cover layer using the Clumpiness Index in Fragstats. The index equals the "proportional deviation of the proportion of like adjacencies involving the corresponding class from that expected under a spatially random distribution" and ranges from -1 to 1 (with 1 being maximally clumped) [54]. We calculated the index for each cell using a 1 km moving window size. We did not include distance to human influence (e.g., road, household) because it was highly correlated with elevation for some pandas and not meaningful for fine-scale analyses since human establishments are all located on the outskirts of the study area, making it difficult to separate from other variables (such as elevation).

To relate these habitat characteristics to habitat use, we plotted the mean use (as predicted from the previous UD estimation procedure) across discrete classes of each of these habitat characteristics. We also examined the relationship between the UD and three of the habitat characteristics commonly used in habitat suitability models for this species (slope, elevation, and forest cover). We calculated the proportion of the area of each animal's UD in different habitat suitability classes according to a four-class habitat suitability scheme outlined by Liu et al. [14], i.e., unsuitable, moderately suitable, suitable and highly suitable. We performed this analysis for both the entire year and for each season. We followed the convention adopted by Schaller et al. [11] to treat April-June as spring, July-October as summer-autumn, and November-March as winter. These seasons roughly correspond to pandas' foraging patterns in this reserve. To further examine habitat use across time, we also plotted the monthly mean of each habitat characteristic among points obtained from the GPS collars (since these points had a specific time period attached to them). To minimize potential bias, we limited data to one randomly chosen point per day for this analysis only.

### Habitat use models

We designed resource utilization functions (RUF) to examine predictors of habitat use within the home ranges of the panda subjects. The RUF approach involves characterizing the relationship between the utilization distribution (UD) of an animal and a set of spatially-explicit habitat characteristics in a regression model [2]. We built both a full model with year-long data and sub-models to represent ecological seasons. For both the full model and sub-models, a separate RUF model was built for each panda individual but model results were later combined for an aggregate analysis.

The RUF model used was a simultaneous autoregressive model (SAR). We chose this model because our data were spatially autocorrelated, violating the assumption of independent observations that is required in an ordinary least squares (OLS) model. The SAR models accounted for spatial autocorrelation in the data by including a non-zero covariance structure to produce more accurate coefficient estimates [55]. The form of the model was as follows:

y=A+ρW(Y−A)+ε

The response variable y was the predicted probability of use obtained from the earlier UD estimation procedure, *W* was a spatial neighbor matrix, *A* was the vector of independent variables related to previously mentioned habitat characteristics, and *ρ* was an interaction parameter indicating the amount of autocorrelation between neighboring points [56]. *ρ* was defined by the inter-point distance over which neighborhood values were spatially autocorrelated [55]. This distance was determined by visual interpretation of semivariograms which showed autocorrelation up to 400 m for all individuals except the male (900 m). The response variables were log_10_- transformed to meet model assumptions. Nonlinear terms (quadratic and cubic) were also included in the models. We tested multiple interaction terms among habitat characteristics but did not include them in the final model because they were not consistently significant across collared individuals. We tested for multicollinearity using the variance inflation factor (VIF) and found no significant multicollinearity (all VIF were <3, well below a cut-off of 5 recommended by [57]). We assessed model fit of the best model by plotting the actual UD to that predicted in the autoregressive model (since R^2^ is not meaningful for this purpose in autoregressive models).

We combined model results for a population-level assessment of resource use using a method outlined by Marzluff et al. [2]. Partial regression coefficients were standardized to account for differences in measurement scale across individuals. Mean standardized regression coefficients were calculated as:

$${\hat{\beta }}_{j}={\hat{\beta }}_{j}^{*}\frac{s_{xj}}{s_{RUF}}$$

where ${\hat{\beta }}_{j}^{*}$ is the partial regression coefficient, *s*_*xj*_ is the standard deviation of the variable measured and *s*_*RUF*_ is the standard deviation of the utilization distribution. We then tested the null hypothesis that each $\hat{\bar{\beta_{j}}}$ differed from 0 using a *t* test.

### Habitat selection models

We assessed the pandas' habitat selection according to a method proposed by Millspaugh et al. [58] as an extension of compositional analysis [3]. In this method, the habitat use of discrete habitat types is compared to the proportion of the habitat types that are available for the animal to choose from. We created 2–6 classes for each habitat variable by dividing the full range of the variable into equal intervals. In cases where one or more pandas did not have any observations in a class at the tail ends of the variable's distribution, we lumped classes together (e.g., we created a category for >50° slope because only a few pandas had slopes above 50°). We summed the UD values (up to the 95% UD) by habitat type to represent proportional use of each type by the animal. We calculated availability as the proportion of area taken up by each habitat type. We included two measures of availability: one calculated over each animal's home range and one including the entire study area (defined by drawing a minimum convex polygon around all panda home ranges and buffering this region by 500 m). These corresponded to within-home range and at-home range selection (second and third order selection by Johnson [59]).

We calculated and plotted the difference between the log-transformed used and available habitats across classes. We used the Wilks' lambda statistic to test for significant selection of each habitat characteristic and subsequently performed a ranking analysis on each habitat class. We ran both analyses using randomization tests (n = 500 runs). We performed an eigenanalysis on selection ratios to test for the assumption of similar selection patterns across individuals (as required for running the previous compositional analysis). All analyses were performed using the R statistical computing software [60] mostly using the "adehabitatHS" package [61].

## Results

### Habitat characteristics across giant panda utilization distributions

**Fig 2:**
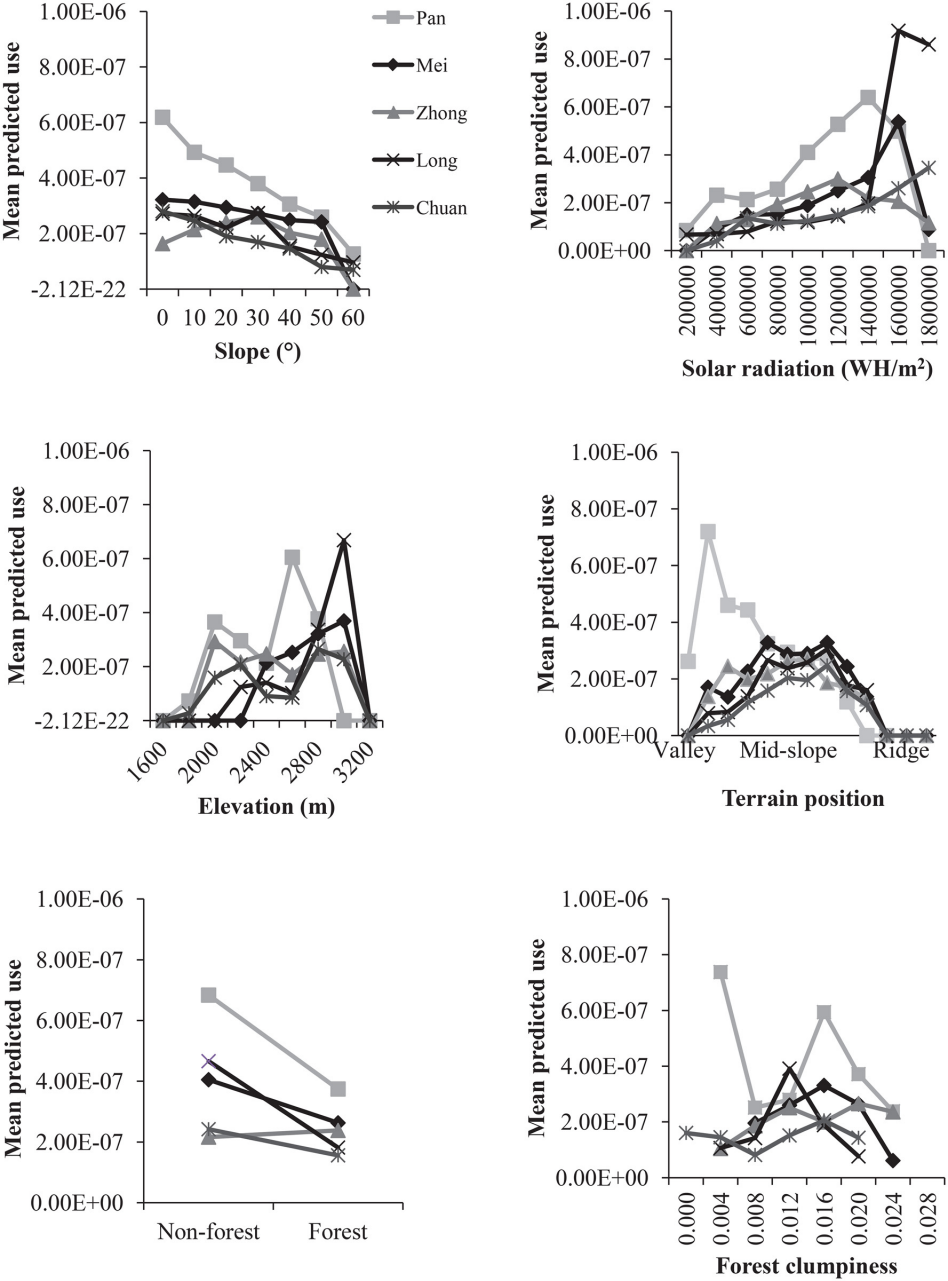
Proportion of giant panda utilization distributions occurring in different classes of habitat characteristics. Chinese names refer to individual pandas.

**Fig 3:**
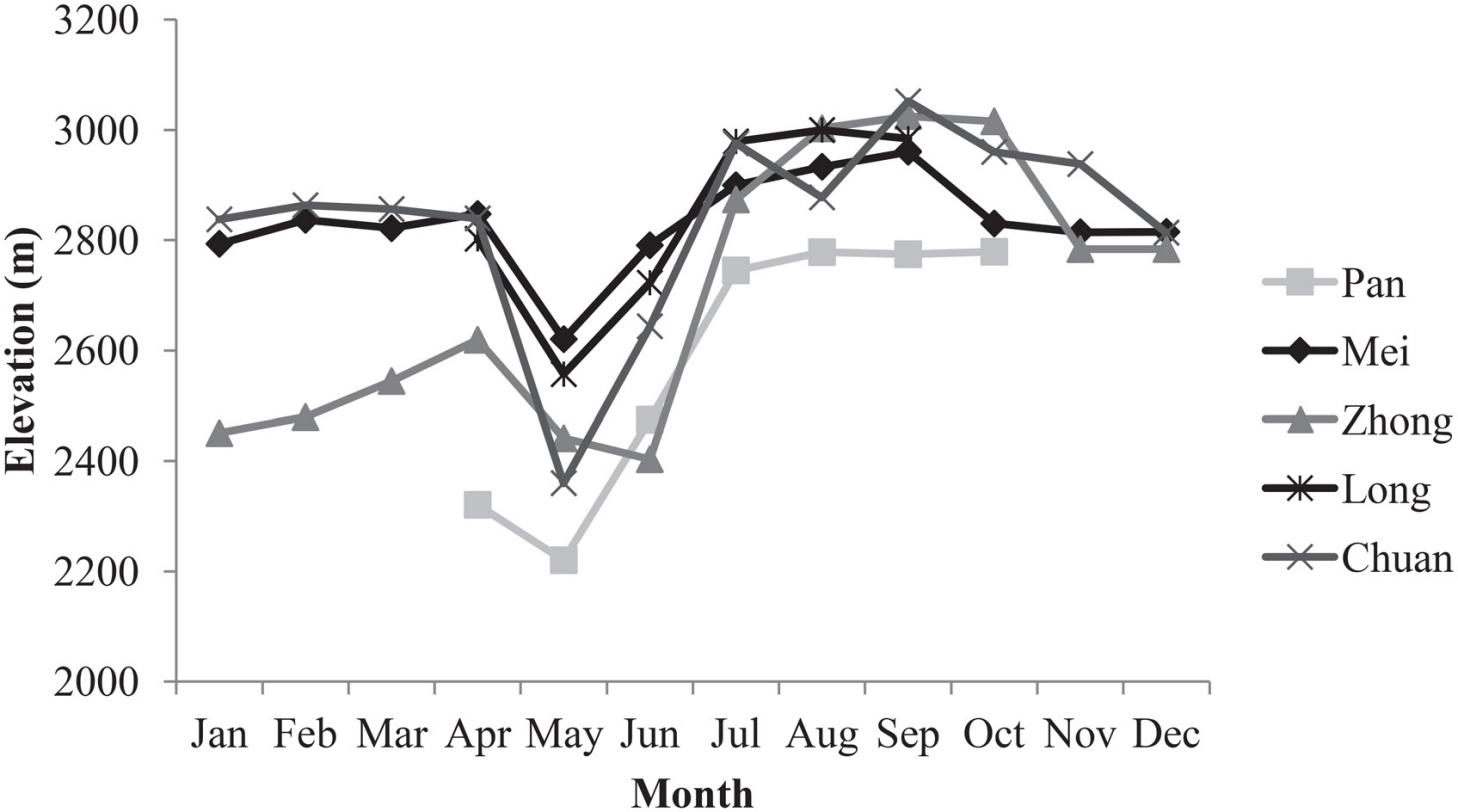
Habitat use of GPS-collared giant pandas with respect to elevation over time. Values shown are the means for each month after randomly selecting one point per day. Chinese names refer to individual pandas.

Pandas used areas with gentle, mid-mountain slopes at middle to higher elevation receiving higher solar radiation (Fig 2). Pandas also used areas of non-forest and moderately clumped forests more intensively than areas that were completely forested. Panda use of elevation varied over time, as pandas migrated from mainly 2200–2600 m in Apr-June to 2800–3000 m for the remainder of the year (Fig 3). There was no consistent difference in habitat use across seasons for the other habitat characteristics.

**Fig 4:**
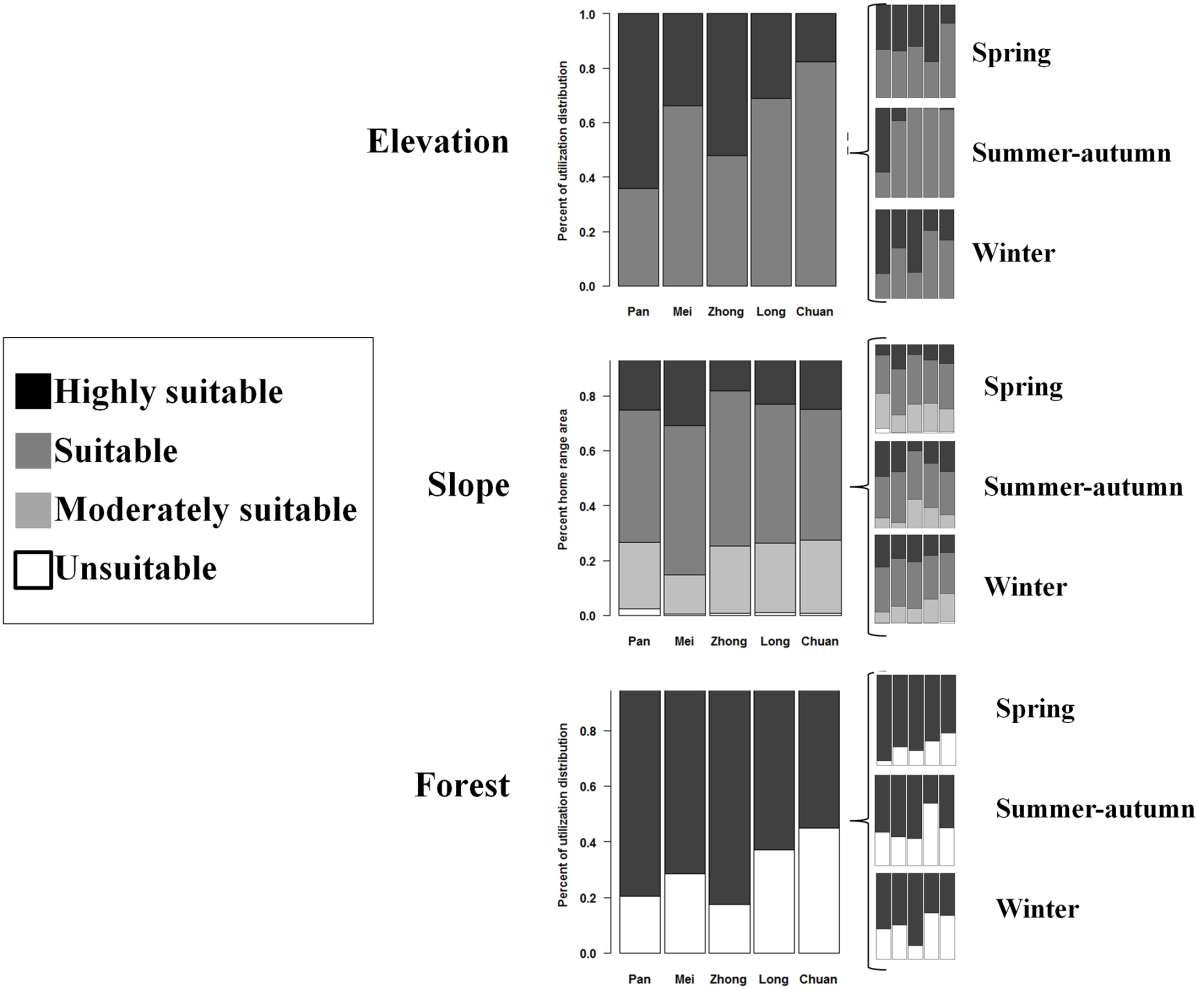
Proportion of GPS-collared panda's utilization distributions in different habitat suitability classes for (a) elevation, (b) forest, and (c) slope. Classes were derived from Liu et al. (1999). Mid-elevations, forest, and gentle sloped areas have higher suitability. The main plots are the entire year and sub-plots on the right are broken down by season.

The majority of the giant pandas' utilization distributions were found within habitats predicted to be suitable habitat using current habitat suitability models for the species (Fig 4). Pandas used elevations completely within highly suitable and suitable elevation ranges (1500–3250 m). However, 18–42% of their utilization distributions were in areas classified as non-forest, a habitat type typically deemed unsuitable for their inhabitance. In addition, 14–26% of their utilization distributions were found in areas previously deemed too steep to be suitable panda habitat (above 30°). The lowest quality habitats were occupied in spring, when pandas were at lower elevations on steeper slopes (albeit in areas with more forest cover, Fig 4).

### Habitat use across space and seasons

**Fig 5:**
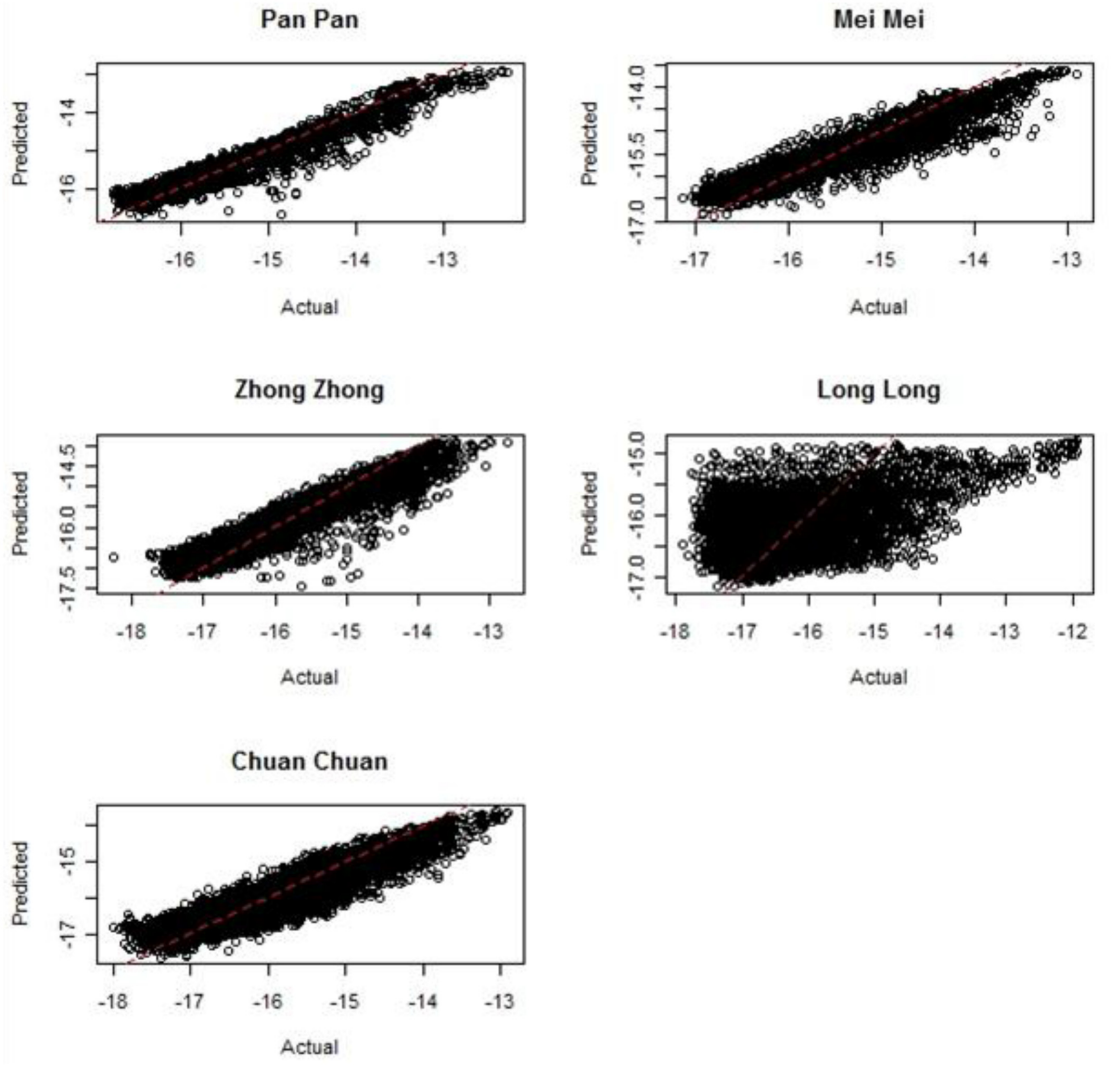
Fit of simultaneous autoregressive (SAR) models depicted by actual versus predicted log-transformed response variables (utilization distributions) for each panda.

In the resource utilization functions models for the entire year, pandas preferentially used areas with intermediate terrain positions, higher solar radiation, higher slope, and non-forest (Table 2). Elevation was negatively related to panda use but the overall pattern was bimodal, as illustrated in Fig 2. When dividing the data up by seasons, solar radiation was significant in spring and winter only (Table 2). Slope and terrain position were only significant in spring. Forest presence was a negative predictor in summer-autumn and winter and forest clumpiness was a positive (nonlinear) predictor in summer-autumn. Across pandas, all models had a significant spatial autocorrelation component (all *α* significant at p<0.01) and were a significantly better fit from an ordinary least squares multiple regression model (ΔAIC > 2). Plots of actual versus predicted use revealed that the fit of the models was generally good, but varied across pandas (Fig 5).

**Table 2:** Contribution of habitat variables to predicting giant panda habitat use across their utilization distributions (n = 5). Only significant variables are shown.

factor	standardized $\;\hat{\bar{\beta }}$	95% CI		P ($\bar{\beta }$ = 0)	# pandas	
					+	-
**Full**						
elevation	-0.32	-0.51	-0.14	<0.01	0	5
slope	0.03	-0.00	0.06	0.06	3	0
TPI	0.10	-0.02	0.22	0.08	4	1
TPI^2^	-0.02	-0.05	-0.00	0.07	0	3
solar	0.09	-0.01	0.19	0.07	4	1
forest	-0.02	-0.04	0.00	0.08	0	3
**Spring**						
slope	0.06	-0.01	0.12	0.08	4	0
TPI	0.08	-0.01	0.18	0.07	3	0
solar	0.13	0.01	0.24	0.04	5	0
**Summer-Autumn**						
elevation	-0.26	-0.41	-0.10	<0.01	0	4
forest	-0.08	-0.15	-0.00	0.04	0	3
clumpiness^3^	0.06	0.00	0.12	0.04	3	0
Winter*						
solar	0.12	0.03	0.20	0.02	2	0
forest	-0.05	-0.12	0.01	0.09	0	1

*only 3 pandas had data available for this season

### Habitat selection for different habitat characteristics at multiple scales

**Fig 6:**
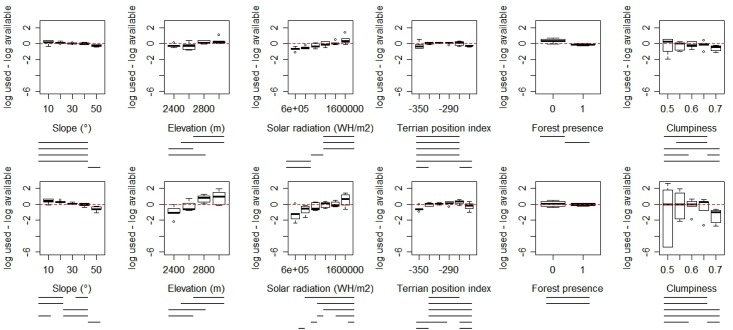
Habitat selection by GPS-collared giant pandas for various biogeophysical characteristics at two selection levels- the within-home range level (top row) and the at-home range level (bottom row). Habitat use of each class was calculated as the proportion of the utilization distribution. Habitat availability was calculated as the proportion of habitat available within individual home ranges (top row) and within the entire study area (bottom row). Lines below each plot represent significant differences in selection across levels determined via randomization tests.

With regard to habitat selection, for the year-long models, there was a marginally statistically significant pattern of overall selection against low terrain position and against the highest clumped forest at the at-home range level (with availability defined as the entire study area; p = 0.07 and p = 0.06, Fig 6). No other overall selection patterns were found but significant effects were seen in several variables for individual classes (e.g. slopes of 40–50°), particularly selection against the lowest solar radiation and steepest slopes (see lines under plots in Fig 6). With regard to seasons, in spring, significant selection was found for non-forest at the within-home range level (with availability defined as limited to each animal's home range; p = 0.02). In summer, significant results were found for selection for gentle slope and higher solar radiation at the within-home range level, selection against low terrain position at the at-home range level, and selection against forest at both selection levels (all p< 0.1). We did not test for significance of selection in the winter models due to the small sample size (n = 3 animals, since collars ceased functioning on the other 2 animals included in the study during this season). The eigenanalysis on selection ratios revealed differences in habitat selection patterns across individuals, suggesting that the significance tests should be interpreted with caution.

## Discussion

This study makes new contributions to understanding habitat use and selection of the endangered giant panda. These include the observation of pandas using a wider range of habitats than previously thought and the illustration of key differences between habitat use and habitat selection for variables such as slope and forest at multiple scales. Although the sample size is small and results should be cautiously interpreted, this is a common challenge for rare and government-protected species facing restrictions on telemetry permits [62, 63]. We were fortunate to be granted special permission by the Chinese government to collar these five individuals after the 11 year ban on collaring pandas and have already been able to draw meaningful and novel findings on space use and human impacts from this unique dataset [23, 64, 65]. Because pandas are an elusive species whose habitat use has mainly been studied at the population level in the past, several new insights can be drawn from this study.

One of the main contributions of the study is the characterization of spatial variation in habitat use by individual giant pandas. The RUF approach allowed us to go beyond the typical binary response variable of used vs. non-used or used vs. available. By modeling panda habitat use across the entire home range, we found that pandas used a wider range of resources than previously appreciated or detected in transect surveys. For instance, moderate to severely steep slopes (over 30°) made up from 14 to 26% of the pandas' utilization distributions. In the past, these sloped areas have been labeled as "marginally suitable" to "unsuitable". This delineation has profound implications for modeling panda habitat over large scales in that it may potentially result in an underestimation of available suitable habitat (see also [20]).

Our study also demonstrated the important distinction between habitat use and habitat selection with respect to topographic slope that has not been made in previous literature. Pandas in fact used steep slopes more than gentle ones. It is likely that pandas used some areas of moderate and steep slopes intensively because they were otherwise valuable to them, potentially due to characteristics of the bamboo stands we did not measure (e.g. age, density). This was especially true in the spring season when pandas migrated down to the lower elevations. This may be because lower elevations tended to have more moderate and steep slopes available (see Fig 5). Spring is also the season when pandas forage on a higher energy food source that is more patchily distributed [11] and thus may be more likely to compromise on habitat use preferences to obtain greater energetic returns from food. Nonetheless, pandas did select against steep slopes with respect to their overall availability in the home range, and to a greater extent, their availability across the whole study area. This avoidance is likely related to inaccessibility of some areas with steep slopes.

Another important observation from this study was that the panda utilization distributions included areas classified as non-forest, which goes against the prevailing understanding of forest being a requirement for panda inhabitance [14]. This finding is likely related to pandas making use of shrublands that still contain bamboo. In fact, forest gaps can promote bamboo growth in otherwise good biophysical conditions due to removal of competition for resources from the overstory [66]. This is corroborated by our finding that pandas used areas with moderately clumped forest as opposed to highly clumped forest areas in summer-autumn. The lack of significance of non-forest for panda habitat in spring could relate to the fact that non-forest at the lower elevations that are occupied by pandas at this time of year may have been formed through different processes than that at higher elevations. Non-forest at low elevations is more likely the result of human disturbance as opposed to naturally-occurring shrublands interspersed with forests at higher elevations, but further research is needed on this topic. Pandas even selected some non-forest pixels in higher proportion than their availability in the home range and study area. Use and selection of forest gaps and shrublands probably depends forest disturbance history, the spatial extent of such areas, and the broader pattern of habitat distributed in the areas surrounding such patches, complexities that require further study.

Results showing pandas used higher solar radiation and selected against lower solar radiation is in keeping with previous literature [17]. Pandas prefer warmer areas because they allow for retention of body heat and thus maximization of thermal energy in their cool habitat areas and/or because warmer areas may also support bamboo growth [17]. Our study adds a new angle to this previously documented phenomenon by demonstrating for the first time the importance of solar radiation in a multivariate model framework.

Our findings on the importance of elevation and the shift in elevational use across seasons are also in keeping with previous literature [11, 24], patterns that relate to the availability of different bamboo species found at different elevations. The importance of elevation was also apparent in the RUF models built by Zhang et al. [24] for four GPS-collared pandas living in the Qinling mountains, where pandas migrate in the opposite direction (upward in elevation) during spring. Also similar to Zhang et al.’s models were our results of gentler slope use during winter relative to spring and preference for higher solar radiation (expressed differently as lower hillshade in Zhang et al.’s models) during the summer. However, previous studies have found differences in some aspects of panda behavior between the Qinling mountains and our study area [67]. Furthermore, some prior studies have also highlighted terrain position as a potentially important variable, but patterns have varied across studies [20]. In our study, avoidance of the valleys is likely related to human impacts that may be more likely to occur in those areas. Therefore, further research (using larger sample sizes) is required to ascertain if there are significant differences in habitat use and selection by panda individuals located in different mountain regions, perhaps due to different environmental conditions.

Further research should also be done on the ground to detect and quantify clandestine human activities such as hunting or herb collection that may occur throughout the habitat and may affect panda habitat use and selection. Livestock grazing is another human impact that may warrant further study [51, 65, 68]. Livestock grazing occurred in one portion of our study area but only overlapped minimally with the study pandas, thus making it difficult to draw robust conclusions about the effect of livestock on pandas using the modeling framework in this study (but see [65]). Future studies conducted on a larger sample size of pandas in a single area might also address the potential effect of competition among neighboring pandas on habitat use and selection. A larger sample size of pandas would also allow for statistical comparisons of habitat use and selection among individuals and as a function of life history characteristics (e.g., sex, age). Our model results suggest that there is high variability among individuals (e.g., only three individuals had significant effects for some variables like slope or forest cover; some variables negatively affected habitat use for some pandas and positively affected habitat use for others). Yet, our sample size does not allow identifying potential sources of this variability. Efforts should also be made to obtain continuous data on bamboo characteristics (e.g. species, cover, age, density) at high spatial resolution across panda home ranges so that data on the panda’s food source can be incorporated into habitat use and selection models. Another area of uncertainty lies in the effect of the accuracy of the estimates of forest cover, slope, and other variables at various spatial scales and how such uncertainty may affect large-scale modeling and management.

Our study has several implications for management of the giant pandas and their threatened habitat. Our findings suggest that steeper habitat and even some non-forest areas previously labeled as unsuitable for the pandas may in fact play important roles in their survival and should not be discounted by managers. Nonetheless, managers should allocate more funding and labor towards repeated on-the-ground field surveys of habitat in different seasons to better determine the value of specific areas for the pandas. Even areas with no visible panda signs may still be used by pandas as part of their home ranges if they are spatially continuous with other valuable panda habitat, making further and more extensive telemetry data invaluable for managers. Such data would improve protected area designations, zoning delineations within protected areas, and corridor designations, therefore improving the conservation status of a broader range of habitat types used by the pandas. Our study also can inform research on habitat use and selection across other species. Our work demonstrates the utility of the RUF approach for species in which a clear understanding of the entire picture of habitat use and selection across the home ranges of individuals is lacking. The significance of spatial autocorrelation in our models was also revealing. This component is often overlooked in RUF models despite the fact that neighboring points are not independent [58]. Finally, our study also demonstrated that establishing a nuanced understanding of habitat use and selection by individual animals across space can be valuable for clarifying models and assumptions made about animal-habitat relationships at broader scales.
